# Differences between the perspectives of physicians and patients on the potential barriers to optimal diabetes control in China: a multicenter study

**DOI:** 10.1186/s12913-018-3783-5

**Published:** 2018-12-12

**Authors:** Chun Liu, Shaoyong Xu, Jie Ming, Aihua Jia, Yingji Wei, Hui Li, Yang Jiao, Mingxi Song, Yadong Zhao, Yafang Du, Wenjuan Yang, Xiaoqiang Lu, Shengqi Shi, Hui Tong, Guangtang Jia, Guohua Zhao, Li Wang, Mei Zhang, Junlin Wang, Wenshu Liu, Lin Fang, Fuhong Dong, Qiuhe Ji

**Affiliations:** 1 Department of Endocrinology, Xijing Hospital, Air Force Medical University, 169 Changle Road West, Xi’an, 710032 China; 2 Department of Endocrinology, Yulin First Hospital, Yulin, 719000 China; 3 School of Nursing, Third Military Medical University, Chongqing, 404100 China; 4 Department of Endocrinology, Shanxi Provincial Peoples Hospital, Xi’an, 710032 China; 5 Department of Endocrinology, The Second Affiliated Hospital of Xi’an Jiaotong University, Xi’an, 710032 China; 6 Department of Endocrinology, The Fourth Hospital of Xi’an, Xi’an, 710032 China; 7 Department of Endocrinology, High-tech Hospital, Xi’an, 710032 China; 8 Department of Endocrinology, Changan Hospital, Xi’an, 710032 China; 9 Department of Endocrinology, Aerospace Hospital, Xi’an, 710032 China; 10 Department of Endocrinology, Huxian County People’s Hospital, Xi’an, 710032 China; 11 Department of Endocrinology, Zhouzhi County People’s Hospital, Xi’an, 710032 China; 12 Department of Endocrinology, The second people’s Hospital of Shenmu county, Yulin, 719000 China; 13 Department of Endocrinology, Fugu County Hospital, Yulin, 719000 China; 14 Department of Endocrinology, Central Hospital of Baoji, Baoji, 721000 China; 15 Department of Endocrinology, Qishan County Hospital, Baoji, 721000 China; 16 Department of Endocrinology, Fengxiang County Hospital, Baoji, 721000 China; 17 Department of Endocrinology, Ankang Central Hospital, Ankang, 725000 China; 18 Department of Endocrinology, Xixiang County Hospital, Hanzhong, 723000 China; 19 Department of Endocrinology, Xunyang County Hospital, Ankang, 725000 China

**Keywords:** Diabetes control, Barriers, Cross-sectional study, China

## Abstract

**Background:**

To investigate the potential barriers to optimal diabetes control by evaluating the different perspectives of physicians and patients on such matters in China.

**Methods:**

This multi-center survey was conducted from December 2015 to March 2016. A multi-stage stratified random sampling method was used to sample representative diabetes physicians and patients in 18 hospitals in Shaanxi province, China. A self-designed questionnaire was used. The questionnaire mainly consisted of 2 questions for physicians and 1 question for patients of which the participants were required to rank in priority of 3 (for physicians) and 2 (for patients) choices from a list of barriers. The strategies to improve diabetes control were only in the questionnaire for physicians.

**Results:**

A total of 85 physicians and 584 patients completed the questionnaire. Physicians and patients differed regarding the patients’ awareness of the risk of diabetes: over 70% of the physicians believed that the patients had no sufficient understanding of the harm and risk of diabetes, whereas the patients believed otherwise. Both physicians and patients considered self-monitoring of blood glucose to be an important link of glucose control; unfortunately, most of the patients failed to do so in practice. In addition, physicians considered “improving health insurance coverage for diabetes” as the first important measure and “providing more and easy-to-use diabetes brochures or educational materials for patients” as the second important measure to improve diabetes control.

**Conclusion:**

The survey revealed differences between the perspectives of physicians and patients on the potential barriers to optimal diabetes control. The main potential barriers to optimal diabetes control were patient’s poor lifestyle interventions, limited understanding of the danger of diabetes, and poor self-monitoring of blood glucose. From the physicians’ perspective, China’s primary focus about diabetes control in the future should still be put on diabetes education, particular the importance of lifestyle interventions.

**Electronic supplementary material:**

The online version of this article (10.1186/s12913-018-3783-5) contains supplementary material, which is available to authorized users.

## Background

Diabetes prevalence has been increasing dramatically in China in recent years. In 2010, the prevalence rate was 11.6% for diabetes and 50.1% for pre-diabetes in adults over 18 in China; the crude prevalence rate was 9.5% for diabetes and 35.5% for pre-diabetes in Shaanxi province; currently, the diabetic patient population in China accounts for approximately one-fourth of the diabetic patients worldwide [1]. With the rise in the overall incidence of diabetes and the incidence of diabetes in younger populations in China, individuals and the society are facing a higher burden of diabetes treatment. Unfortunately, even with continued innovation in anti-diabetic drugs and constantly evolving ideas regarding diabetic treatment, a substantial increase in expenses for diabetes care has failed to significantly improve the diabetes control rate; conversely, the morbidity of diabetes has increased each year [2]. Therefore, it is important to investigate the potential barriers to optimal diabetes control.

Diabetes physicians have gained rich experience in the daily diagnosis and treatment of diabetes, and therefore, their feedback on the potential barriers to optimal diabetes control is extremely valuable. Numerous Chinese and foreign studies have shown that from the perspective of physicians, the patient’s lifestyle [3, 4], mental health [5], economic status [6], and religion [7] have a significant effect on diabetes control; furthermore, the physician’s vocational education [8], specialist nurses [9], and education [10] also play important roles in diabetes control. However, the opinions differ between physicians in different surveys [3–6]. Notably, few such studies in the past have investigated the potential barriers to optimal diabetes control from the perspective of patients; consequently, an understanding of different perspectives between physicians and patients will, undoubtedly, provide an important reference for improving diabetes control and patient compliance in the future.

As such, this multi-center study was designed to investigate the potential barriers to optimal diabetes control by evaluating the different perspectives of physicians and patients on such matters, and also investigate the physician-recommended public health measures for improving diabetes control in China.

## Methods

### Study design

**Fig. 1:**
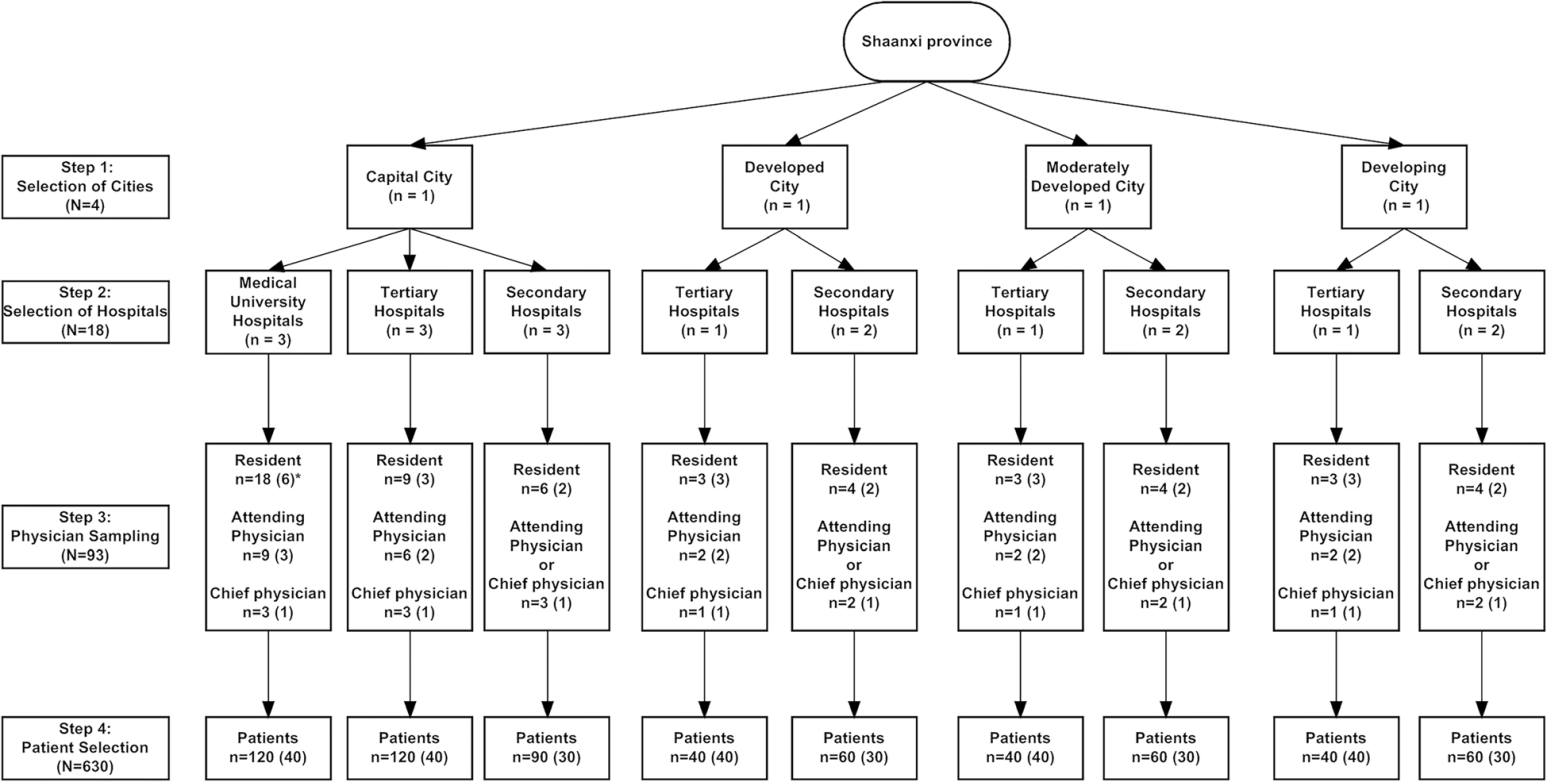
Protocol design flowchart. *: numbers in each hospital

This multi-center cross-sectional survey was conducted from December 2015 to March 2016. A multi-stage stratified random sampling method was used to sample representative diabetes physicians and patients in Shaanxi province, China for this survey (Fig. 1).

Step 1 involved sampling physicians and patients in the cities. In addition to the capital city (Xi’an), physicians and patients from an economically developed prefecture-level city (Yulin, annual Gross National Products GDP > 50,000 yuan per capita), a moderately developed prefecture-level city (Baoji, annual GDP 30,000–50,000 yuan per capita), and a developing prefecture-level city (Ankang, annual GDP < 30,000 yuan per capita) were sampled; the GDP was classified according to the 2015 GDP in Shaanxi Province. This step was non-randomized. Step 2 involved stratifying the hospitals in the capital city and the three prefecture-level cities. The hospitals in the capital city were stratified into medical university hospitals, tertiary municipal hospitals, and secondary county hospitals; the hospitals in the prefecture-level cities were stratified into tertiary municipal hospitals and secondary county hospitals. Step 3 involved sampling the hospitals. Given the differences in the city sizes and the distribution of medical resources between the capital city and the prefecture-level cities, we used a simple random sampling (drawing) method to randomly sample three medical university hospitals, three tertiary municipal hospitals, and three secondary county hospitals in the capital city in addition to one tertiary municipal hospital and two secondary county hospitals in each of the prefecture-level cities. Thus, we sampled a total of 18 hospitals, including three medical university hospitals, six tertiary municipal hospitals, and nine secondary county hospitals. Step 4 involved sampling the physicians. According to the distribution of physicians in the different levels of the hospitals, we randomly sampled six residents, three attending physicians, and one chief physician from each medical university hospital; three residents, two attending physicians, and one chief physician from each tertiary municipal hospital; and two residents and one attending or chief physician from each secondary county hospital. Thus, we sampled a total of 93 physicians, including 54 residents, 21 to 31 attending physicians, and nine to 19 chief physicians. Step 5 involved sampling the patients. We used the cluster random sampling method and enrolled all of the patients with type 2 diabetes (based on World Health Organization 1999 criteria) who were hospitalized for diabetes treatment in department of endocrine during a certain time window and who met all of the study criteria, had poor diabetes control (HbA1c ≥ 7%), and were willing to participate in this study, until the planned number of participants was reached. Given the difference in ward capacity between the different levels of hospitals, we planned to sample 40 patients in each medical university hospital and tertiary municipal hospital and 30 patients in each secondary county hospital, for a total of 630 patients, which provided a sufficient sample according to previous literatures [11–23].

### Data collection

We developed our own questionnaire for data collection. While designing the questionnaire, we referred all the barriers and strategies from previous related literatures, and expanded some potential ones localized for the Chinese, which were from a small-scale pre-survey [11–23]. Two versions of the survey were used: the physician version and the patient version. (Additional file 1).

The physician questionnaire had two parts. Part 1 was used to collect basic information on the physician, including title and the type and location of the hospital. Part 2 contained two questions. Question 1 asked “According to your own clinical experience, what do you think are the top three potential barriers to optimal diabetes control?” There were three items could be chosen from a list of eight items. The physicians were asked to rank the factors in the order of importance. Question 2 asked “From the perspective of the government/community/hospital/physician, what do you think are the top three areas that require urgent improvement in order to improve diabetes control?” There were three items could be chosen from a list of nine items. Again, the physicians were asked to rank the areas in the order of importance.

The patient questionnaire contained two parts. Part 1 was used to collect patient demographic data, lifestyle, and history of diabetes. Part 2 contained only one question: “What do you (the patient) think are the causes of your uncontrolled blood glucose?” This was a multi-choice question and the patients could choose more than one factors for uncontrolled glucose. The patients were asked to mark the most important item. See the annex for the questionnaire design and baseline information.

### Statistical analysis

EpiData software (version 2.3) was used to enter the data obtained into the database, and the Statistical Package for Social Sciences 20.0 (SPSS Inc., Chicago, IL, USA) was used for the descriptive statistics. The measurement data were expressed as the numerical mean and standard deviation. Count data are expressed as percentages (%).

## Results

### Physicians questionnaire

A total of 93 physicians were sampled and asked to participate, of whom 85 agreed to participate and complete the physician questionnaire (response rate: 91.4%), including 42 (49.4%) residents, 31 (36.5%) attending physicians, and 12 (14.1%) chief physicians; 30 (35.3%) physicians were from medical university hospitals, 35 (41.2%) were from tertiary municipal (non-teaching) hospitals, and 20 (23.5%) were from secondary county hospitals; 45 (52.9%) physicians were from the capital city, 24 (28.2%) were from prefecture-level cities, and 16 (18.8%) were from counties or lower level cities (Table 1).

**Table 1:** Baseline information of the physicians

Variable	*N* = 85
Title, n (%)	
Resident	42 (49.4)
Attending Physician	31 (36.5)
Chief Physician	12 (14.1)
Hospital, n (%)	
Medical university hospital	30 (35.3)
Tertiary non-teaching hospital	35 (41.2)
Secondary hospital	20 (23.5)
Location, n (%)	
Capital city	45 (52.9)
Prefecture-level city	24 (28.2)
County and below	16 (18.8)

**Fig. 2:**
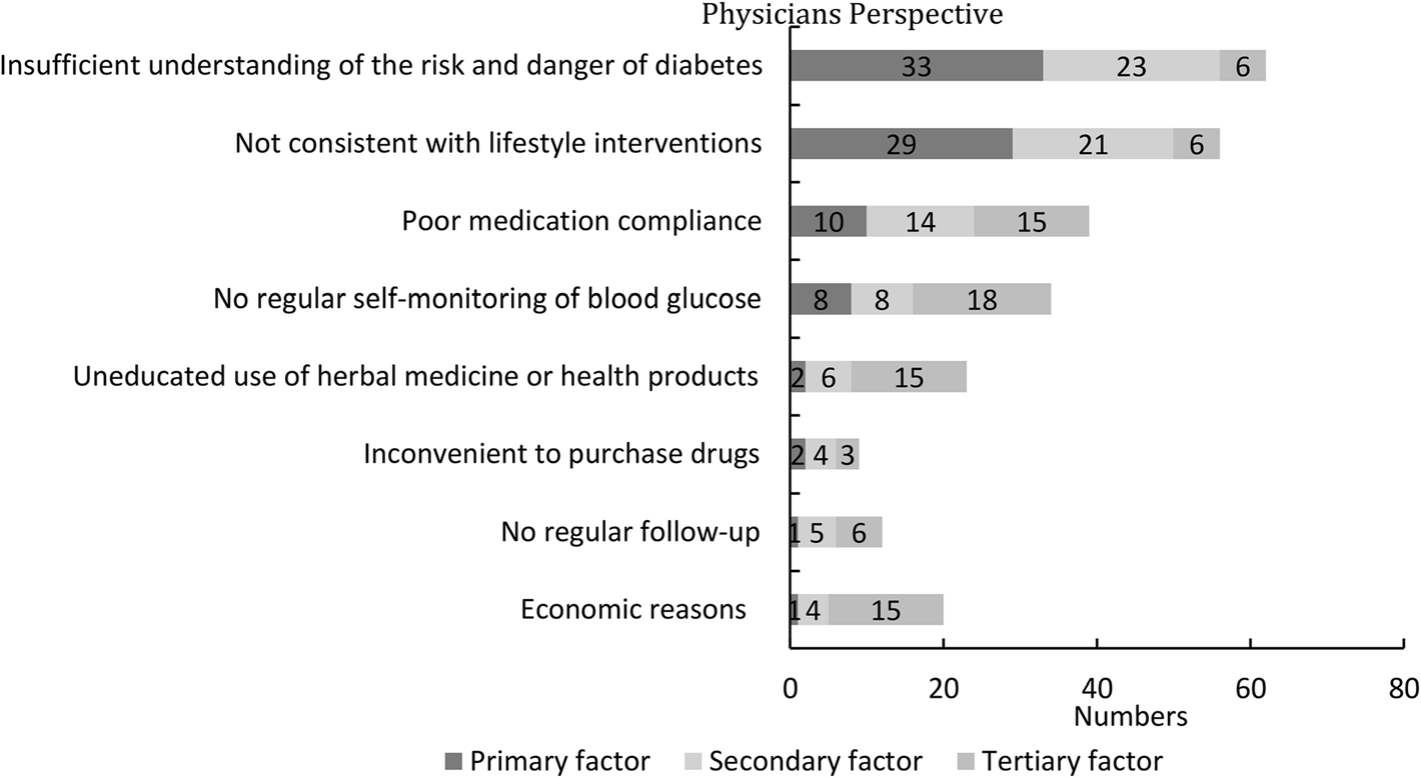
Potential barriers to optimal diabetes control in physician’s perception

For question 1 (Figs. 2), “According to your own clinical experience, what do you think are the top three potential barriers to optimal diabetes control?”, 62 (62/85 72.9%) physicians considered patient’s “insufficient understanding of the danger of diabetes” as an important factor, of whom 33 (33/85 38.8%) physicians ranked it as the most important factor, 23 (23/85 27.1%) physicians ranked it as the second most important factor, and 6 (6/85 7.1%) physicians ranked it as the third most important factor.

In addition, 56 (56/85 65.9%) physicians considered patient’s “lack of perseverance to stick to lifestyle intervention (if even understanding the danger of diabetes)” as an important factor, of whom 29 (29/85 34.1%) physicians ranked it as the most important factor, 21 (21/85 24.7%) physicians ranked it as the second most important factor, and 6 (6/85 7.1%) physicians ranked it as the third most important factor.

Moreover, the physicians considered patient’s failure to monitor blood glucose regularly, poor medication compliance, economic reasons, and the uneducated use of herbal medicine or health products as moderately effective potential barriers to optimal diabetes control. Whereas physicians considered factors that failure to attend scheduled follow-ups or inconvenience to purchase drugs as lower effective factors to diabetes control than other factors.

**Fig. 3:**
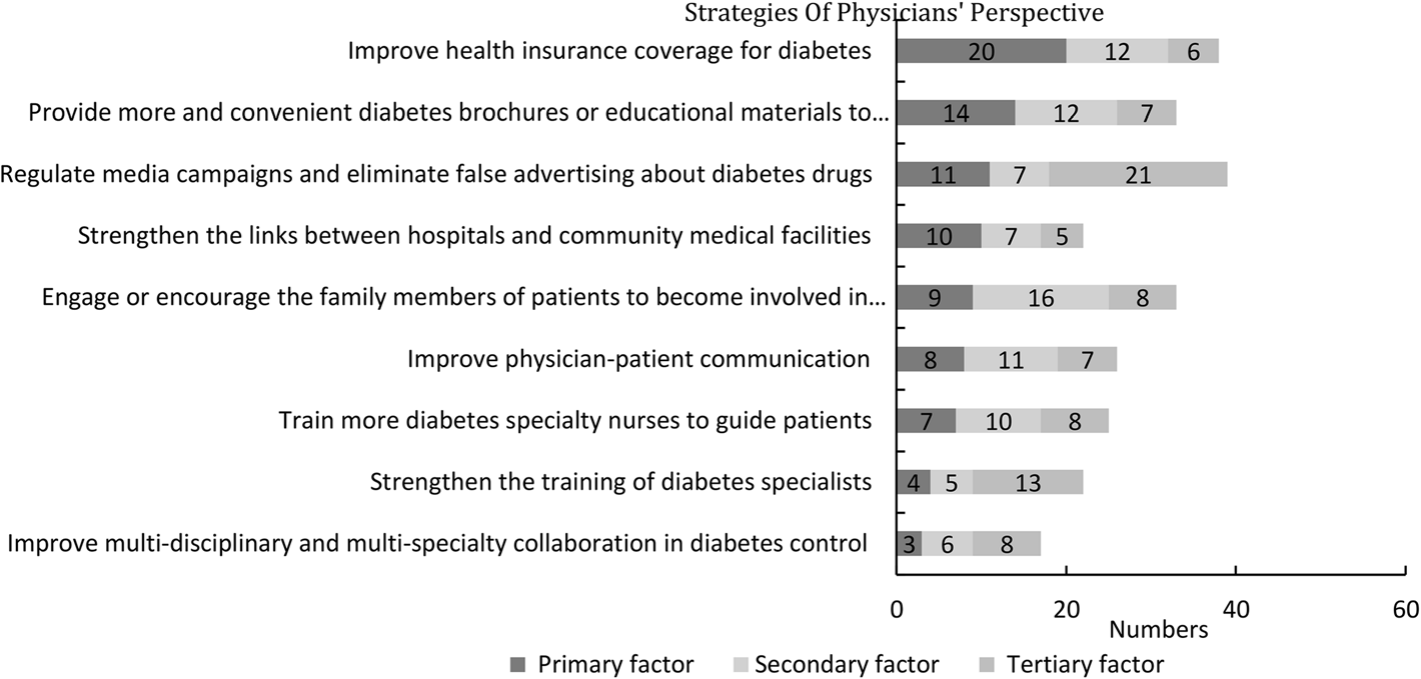
Physician-recommended public health measures for improving diabetes control

For question 2 (Figs. 3), “From the perspective of the government/community/hospital/physician, what do you think are the top three areas that require urgent improvement in order to improve diabetes control?”, 38 (38/85 44.7%) physicians considered “improving health insurance coverage for diabetes” as an important measure, of whom 20 (20/85 23.5%) physicians ranked it as the most important factor, 12 (12/85 14.1%) physicians ranked it as the second most important factor, and 6 (6/85 7.1%) physicians ranked it as the third most important factor.

In addition, 33 (33/85 38.8%) physicians considered “providing more and easy-to-use diabetes brochures or educational materials for patients” as an important factor, of whom 14 (14/85 16.5%) physicians ranked it as the most important factor, 12 (12/85 14.1%) physicians ranked it as the second most important factor, and 7 (7/85 8.2%) physicians ranked it as the third most important factor.

Moreover, although the physicians considered regulating media campaigns and minimizing false advertising, in addition to engaging or encouraging family members to become involved in the care of diabetic patients as important measures for strengthening public health management of diabetes, few ranked these factors as the most important factor. Furthermore, the physicians did not consider factors such as strengthening the training of diabetes specialists, training more specialist diabetes nurses to provide guidance for patients, and improving multidisciplinary and multi-specialty collaboration for diabetes control as urgent public health measures for improving diabetes control.

### Patient questionnaire

A total of 630 patients were sampled and asked to participate, of whom 584 patients agreed to participate and completed the patient questionnaire (response rate: 92.70%). Of the patients who completed the questionnaire, 325 patients were men with a mean age of 56.27 years (± 13.19), and 254 patients were women with a mean age of 58.06 years (± 12.79). Moreover, 242 (44.2%) patients had been diagnosed with diabetes for less than 5 years, 175 (32.0%) patients had been diagnosed with diabetes for 5 to 10 years, and 130 (23.8%) patients had been diagnosed with diabetes for ≥10 years (Table 2).

**Table 2:** Baseline information of the patients

Variable	*N* = 584
Gender, n (%)	
Male	325 (56.0)
Female	254 (43.8)
Mean age, years	57.01 ± 13.27
Male	56.27 ± 13.19
Female	58.06 ± 12.79
Educational level, n (%)	
Primary school and below	165 (31.3)
Middle and high school	226 (42.9)
College and above	132 (25.0)
Economic condition^a^, n (%)	
Poverty and below	79 (13.5)
Subsistence level	266 (45.5)
Well-to-do and above	194 (33.2)
Duration of diabetes, n (%)	
< 5 years	242 (44.2)
5 to 10 years	175 (32.0)
≥ 10 years	130 (23.8)

^a^Economic condition was categorized according to per capita annual net income of households: poverty and below < 5000 China Yuan (CNY), subsistence level 5000–80,000 CNY, and well-to-do and above > 80,000 CNY

**Fig. 4:**
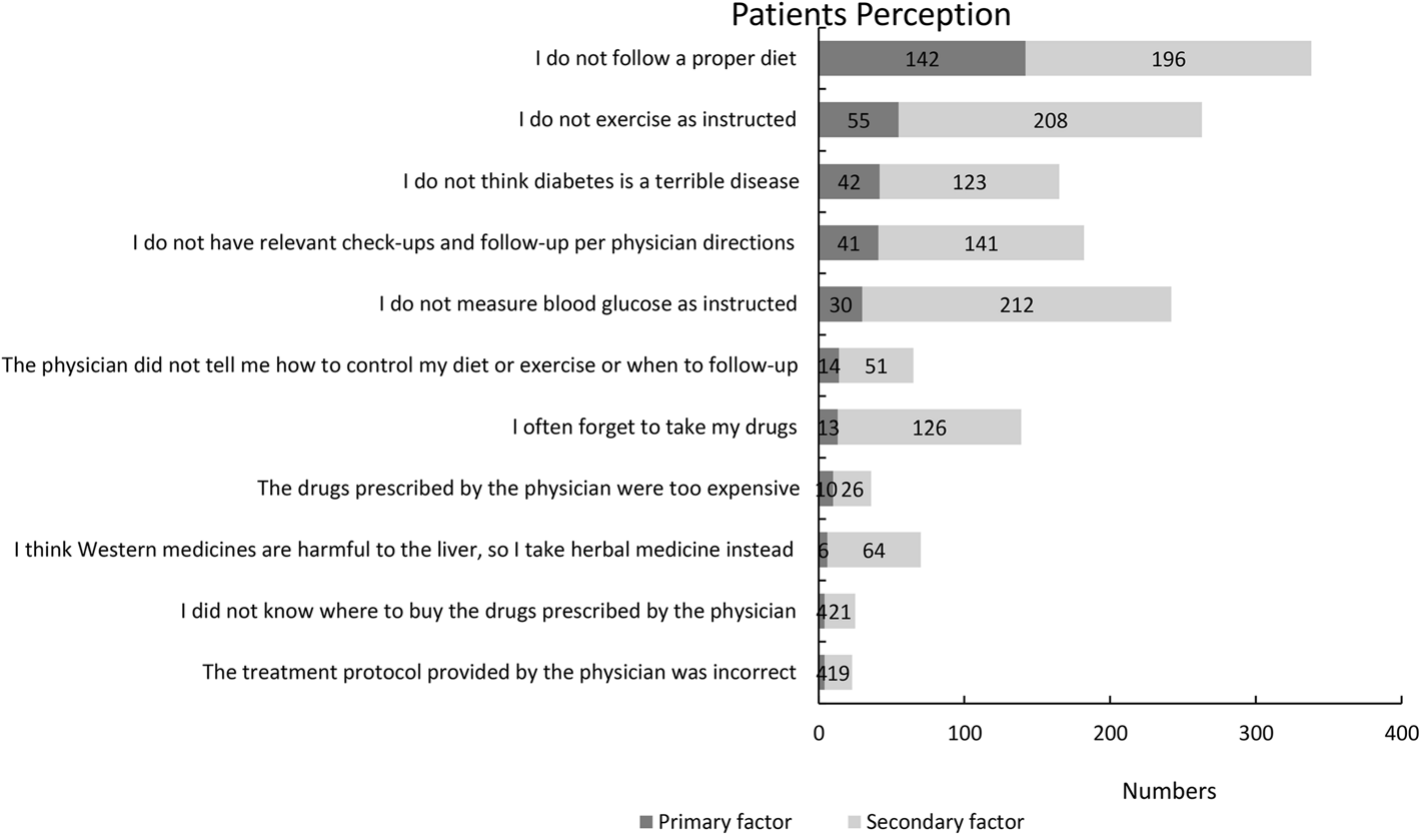
Potential barriers to optimal diabetes control in patient’s perception

The data analysis of the 584 valid patient questionnaires showed that for the question, “What do you (the patient) think are the potential barriers to optimal diabetes control?” (Figs. 4), 338 (338/584 57.9%) patients considered “I do not follow a proper diet” as an important barrier, of whom 142 (142/584 24.3%) patients ranked it as the most important reason and 196 (196/584 33.6%) patients ranked it as the second most important barrier. A total of 263 (263/584 45.0%) patients considered “I do not exercise as instructed” as an important reason, of whom 55 (55/584 9.4%) patients ranked it as the most important barrier and 208 (208/584 35.6%) patients ranked it as the second most important barrier.

Moreover, the patients considered failure to monitor blood glucose as instructed as an important potential barrier to optimal diabetes control. However, the patients did not consider high drug cost, inconvenience to purchase drugs, the use of herbal medicine, and incorrect treatment protocol as potential barriers to optimal diabetes control.

## Discussion

This multi-center cross-sectional study showed that both physicians and patients considered lifestyle as one of the most important potential barriers to optimal diabetes control. However, physicians and patients differed regarding the patients’ awareness of the risk of diabetes: over 70% of the physicians believed that the patients had no sufficient understanding of the harm and risk of diabetes, whereas the patients believed otherwise. In addition, this study showed that both physicians and patients considered self-monitoring of blood glucose to be an important link of glucose control; unfortunately, most of the patients failed to do so in practice.

First, this survey showed that both physicians and patients considered lifestyle as one of the most important potential barriers to optimal diabetes control. “Lifestyle intervention” ranked second in the physician survey, but nearly 70% of the physicians considered it an important factor, suggesting that most physicians recognized its importance, which was consistent with survey results in The United States of America (US) [13], the Middle East, Thailand and South Africa [6, 15]. The patient survey showed that nearly 100% of the patients with poor diabetes control checked “I do not follow a proper diet” or “I do not exercise as instructed” or both, indicating that most Chinese diabetic patients recognized the importance of lifestyle intervention in diabetes control but did not stick to it [21], which is common in both China and other countries [12, 19]. Both physicians and patients considered lifestyle intervention an important factor, highlighting its importance in poor diabetes control in China. In terms of measures for this issue, physicians suggested “providing more and easy-to-use diabetes brochures or educational materials for patients” and “engaging or encouraging family members to become involved in the care of diabetic patients”, which differed from the results in some developed countries. For example, some physicians in the US believed that comprehensive intervention was more important than providing more education materials [18] because in developed countries, patients already had access to basic health education and were generally well-informed. Innovation in drug research and development and the use of insulin could not substitute for the role of lifestyle interventions in improving diabetes control; conversely, a long-term poor lifestyle had adverse effects on drug and insulin therapy [20]. Therefore, we suggest that in Shaanxi Province, which displays uneven development, priority should be given to educational diabetes materials highlighting basic treatment. Meanwhile, active patient engagement [10] and effective self-management skills [14] are important for improving the disease control rate; thus, physicians should encourage and help the family members of patients become involved in diabetes monitoring and control.

Second, physicians and patients differed in the patients’ awareness of the risk of diabetes. Over 70% of the physicians believed that patients had no sufficient understanding of the harm and risk of diabetes, which was consistent with the results in studies conducted in the Middle East, South Korea, and Japan in which physicians believed that patients should learn more about diabetes [16–18]. The importance of diabetes education has been established in the medical community [14, 21]. However, just under 30% of the patients considered “I do not think diabetes is a terrible disease” as an important reason for their poor diabetes control (although this factor ranked third in the patient survey), suggesting that the remaining 70% of the patients may have recognized the danger of diabetes. Thus, physicians and patients differed in their opinion in this regard. Given the physician’s professional background in endocrinology, we would place more confidence in the physician’s opinion. We believe that most of the patients did not have a sufficient understanding of diabetes, which may have impeded diabetes control; moreover, most of the patients may not have a strong interest in developing a thorough understanding of diabetes [18]. Therefore, we conducted a supplementary survey in a subgroup of 50 patients (Additional file 2). The questionnaire had two parts. Part 1contained basic information. Part 2 contained eight questions mainly about the diabetes complications (Question2-Question9). Patients got one score when they know one question in part 2. The results showed that among the 41 patients who rated themselves as someone with an understanding of the danger of diabetes, only 51.2% answered all of the questions correctly, and 66% answered 75% of the questions correctly. Although the patients who believed themselves to understand the danger of diabetes scored higher (mean score) than those who did not (6.78 vs 4.11, *p* = 0.028), they had a limited understanding of the acute and chronic complications of diabetes (Additional file 2). Thus, based on the measures suggested by the physicians during the survey, we recommend using more detailed educational materials to improve patient understanding of the danger of diabetes.

Moreover, we found that both physicians and patients (up to 40%) considered self-monitoring of blood glucose as an important link of glucose control; specifically, “failure to monitor blood glucose regularly” ranked fourth in the physician survey and third in the patient survey as a factor for poor diabetes control. Economic conditions and (long-term) pain during testing were the two main reasons patients cited for failing to monitor their blood glucose regularly based on our clinical experience. The physicians suggested that health care coverage must first be expanded, particularly to cover test strips and needles used for glucose monitoring, similar to situations in some developing countries [11]. Prof. Weiping Jia (China) has also called on the Chinese government to expand health care coverage to cover the costs of glucose monitoring.

In addition, we found that false advertising and certain herbal medicines and health products were important potential barriers to optimal diabetes control in Chinese diabetic patients, suggesting that patients generally had a poor understanding of diabetes and urgently required truthful, accurate, and professional diabetes education. The physicians believed that regulating media campaigns and eliminating false advertising is one of the four public health measures necessary to improve the diabetes control rate. Herbal medicines and health products are unregulated in China, and the abuse of these products affected glucose control in 10% of the patients based on this survey. In addition, we did not include self-evaluation items in the physician survey, and therefore, we were unsure about the effect of iatrogenic events on patients’ glucose control; however, the patient survey showed that iatrogenic events were not a major factor for uncontrolled glucose. Other factors, such as improving physician-patient communication, training of specialty nurses, and regular follow-ups, were considered important factors for improving glucose control.

The study design was well balanced. Based on the distribution of medical resources, we stratified and randomly sampled 18 representative hospitals of different levels in Shaanxi Province. However, this study had some limitations: Firstly, this study was conducted in only one province, and thus, the results may not be applicable to the whole of China. Secondly, the physician’s professional skills were unknown, and thus, we were unable to evaluate the effect of physicians’ professional skills on uncontrolled glucose. Thirdly, factor on economic reasons can be many, so the specific factor may not be available from the results. Fourthly, As the questionnaire only sought physician’s perspective without patients’ perspective for strategies of improvement, hence strategies as suggested by physicians may not be actually effective in improving patient’s diabetes control. Lastly, we only included patients with type 2 diabetes, so the conclusion of this survey may not be generalized to other diabetic population. For example, accessing and maintaining contact with diabetes care services may be the most potential barrier to optimal diabetes control in young patients with type 1 diabetes [24].

## Conclusions

This was the first formal large-scale multi-center survey of diabetes perception in physicians and patients in China. The survey revealed differences between the perspectives of physicians and patients on the potential barriers to optimal diabetes control, and the results showed that the main potential barriers to optimal diabetes control were patient’s poor lifestyle interventions, limited understanding of the danger of diabetes, and poor self-monitoring of blood glucose. Thus, this study suggests that, as physicians and public health agencies, our primary focus about diabetes control in the future should still be put on diabetes education, particular the importance of lifestyle interventions and the danger of diabetes, by developing and promoting more educational materials. In addition, we hope that the Chinese government and society will expand health insurance coverage and take effective measures to regulate media campaigns.

## Additional files

Additional file 1: Survey of the potential barriers to optimal diabetes control. (DOCX 30 kb)

Additional file 2: Survey of patients’ perspectives for diabetes dangers. (DOCX 28 kb)

## Abbreviations

GDP: Gross Domestic Product
HbA1c: HemoglobinA1c
US: The United States of America
