# Supplementary material for: Differences between the perspectives of physicians and patients on the potential barriers to optimal diabetes control in China: a multicenter study
Source: BMC Health Serv Res. 2018 Dec 12;18:961. doi: 10.1186/s12913-018-3783-5 (PMC6292153; doi:10.1186/s12913-018-3783-5)
Supplement: Supplementary file 1 — Survey of the potential barriers to optimal diabetes control. (DOCX 30 kb) [file 12913_2018_3783_MOESM1_ESM.docx]

**Additional file 1**

| Survey of factors for uncontrolled diabetes (physician version) | | | | | | |  |
| --- | --- | --- | --- | --- | --- | --- | --- |
| This anonymous survey is one page long and may take about five minutes to complete. Thank you for your collaboration in this study. Please complete the survey independently. | | | | | | |  |
| Your title: | 🞎 Resident 🞎 Attending physician 🞎 (Associate) chief physician | | | | | |  |
| Your hospital: | 🞎 Medical university hospital 🞎 Tertiary non-teaching hospital 🞎 Secondary hospital | | | | | |  |
| Hospital location: | 🞎 Capital city 🞎 Prefecture-level city 🞎 Suburban, county, township, town, village | | | | | |  |
| On the basis of your clinical experience, what do you think are the top patient factors for uncontrolled diabetes?(Please check only *three* items, and rank them using the numbers *1-2-3*, with *1* as the most important factor) | | | | | | | |
| 1. Economic reasons. | | | | [ ] | | | |
| 2. Inconvenient to purchase drugs. | | | | [ ] | | | |
| 3. Not consistent with lifestyle interventions. | | | | [ ] | | | |
| 4. Insufficient understanding of the risk and danger of diabetes. | | | | [ ] | | | |
| 5. No regular follow-up. | | | | [ ] | | | |
| 6. Poor medication compliance. | | | | [ ] | | | |
| 7. Uneducated use of herbal medicine or health products. | | | | [ ] | | | |
| 8. No regular self-monitoring of blood glucose. | | | | [ ] | | | |
| From the perspective of the government/community/hospital/physician, what do you think are the top three areas that require improvement in order to improve diabetes control?(Please check only *three* items, and rank them using the numbers *1-2-3*, with *1* as the most important factor) | | | | | | | |
| 1. Provide more and convenient diabetes brochures or educational materials to patients. | | | | [ ] | | | |
| 2. Train more diabetes specialty nurses to guide patients. | | | | [ ] | | | |
| 3. Improve health insurance coverage for diabetes. | | | | [ ] | | | |
| 4. Improve physician-patient communication. | | | | [ ] | | | |
| 5. Improve multi-disciplinary and multi-specialty collaboration in diabetes control. | | | | [ ] | | | |
| 6. Strengthen the links between hospitals and community medical facilities. | | | | [ ] | | | |
| 7. Engage or encourage the family members of patients to become involved in diabetes care. | | | | [ ] | | | |
| 8. Strengthen the training of diabetes specialists. | | | | [ ] | | | |
| 9. Regulate media campaigns and eliminate false advertising about diabetes drugs. | | | | [ ] | | | |
| Survey of clinical diagnosis and treatment | | | | | | | |
| 1. Have you received special training on diabetes guidelines (internal department training or external seminars)? | | 🞎 | Yes | | 🞎 | no | |
| 2. Do you think you can perform clinical diagnosis and treatment according to the diabetes guidelines? | | 🞎 | Yes | | 🞎 | no | |
| 3. Are you able to routinely give lifestyle and/or diet advice to new diabetic patients? | | 🞎 | Yes | | 🞎 | no | |
| 4. Are there specialized nutritionists or diabetes nurses involved in your daily practice? | | 🞎 | Yes | | 🞎 | no | |
| 5. Are you able to routinely pay attention to and intervene in the mental stress of new diabetic patients? | | 🞎 | Yes | | 🞎 | no | |
|  |  |  |  |  |  |  |  |

| **Survey of factors for uncontrolled diabetes (patient version)** | | | | | |
| --- | --- | --- | --- | --- | --- |
| **Basic information** | | | | | |
| Name _________________ | | Gender □ Male □ Female | | Age ______ Years | |
| Educational level | □ Primary school and below | | □ Middle and high school | | □ College and above |
| Occupation | □ Responsible person at government agency, enterprise, or public sector institutions | | □ Clerk and associated personnel | | □ Professional and technical personnel |
|  | □ Production personnel in agriculture, forestry, animal husbandry, fisheries, or water conservancy industry | | □ Personnel in commercial and service industry | | □ Soldier |
|  | □ Operation and associated personnel of manufacturing or transport equipment | | □ Not otherwise specified | |  |
| Economic condition | □ Extreme poverty | | □ Poverty | | □ Subsistence level |
|  | □ Well-to-do | | □ Affluent | | □ Extremely affluent |
| Smoking (regular smoking, 1 or more cigarettes per day in the past three months) | | | | □ Yes □ No | |
| Drinking (beer [350 ml or more] or white liquor [50 g or more], three or more times per week in the past three months) | | | | □ Yes □ No | |
| Exercise (30 minutes or more, three or more times per week in the past three months) | | | | □ Yes □ No | |
| Diet (diabetes diet per physician directions or at least not over-eating) | | | | □ Yes □ No | |
| Diabetes history__________ Years | | | |  | |
|  |  |  |  |  |  |

| Status of diabetes control and related reasons | |
| --- | --- |
| How would you rate your glucose control? □ good □ not good If "good", please skip the questions below. | |
| Check the items that, in your opinion, are factors for uncontrolled glucose (√), and choose the one you think is the most important (mark with ★): | |
| 1. The treatment protocol provided by the physician was incorrect. | [ ] |
| 2. The physician did not tell me how to control my diet or exercise or when to follow-up. | [ ] |
| 3. The drugs prescribed by the physician were too expensive. | [ ] |
| 4. I did not know where to buy the drugs prescribed by the physician. | [ ] |
| 5. I do not follow a proper diet. | [] |
| 6. I do not exercise as instructed. | [ ] |
| 7. I do not think diabetes is a terrible disease. | [ ] |
| 8. I do not have relevant check-ups and follow-up per physician directions. | [ ] |
| 9. I often forget to take my drugs. | [ ] |
| 10. I think Western medicines are harmful to the liver, so I take herbal medicine instead. | [ ] |
| 11. I do not measure blood glucose as instructed. | [ ] |
| 12. Other, please specify: |  |
|  |  |

Number: ____
