# Supplementary material for: Differences between the perspectives of physicians and patients on the potential barriers to optimal diabetes control in China: a multicenter study
Source: BMC Health Serv Res. 2018 Dec 12;18:961. doi: 10.1186/s12913-018-3783-5 (PMC6292153; doi:10.1186/s12913-018-3783-5)
Supplement: Supplementary file 2 — Survey of patients’ perspectives for diabetes dangers. (DOCX 28 kb) [file 12913_2018_3783_MOESM2_ESM.docx]

**Additional file 2**

To investigate patients’ perspectives for diabetes dangers, we conducted a supplementary survey in a subgroup of 50 patients in Xijing Hospital between May 1 and June 30 in 2016. The questionnaire had two parts. Part 1 contained basic information. Part 2 contained eight questions mainly about the diabetes complications (Question2-Question9). Patients got one score when they choose YES in each question in part 2. The results showed that among the 41 patients who rated themselves as someone with an understanding of the danger of diabetes, only 51.2% answered all of the questions correctly, and 66% answered 75% of the questions correctly. Although the patients who believed themselves to understand the danger of diabetes scored higher (mean score) than those who did not (6.78 vs 4.11, *p* = 0.028), they had a limited understanding of the acute and chronic complications of diabetes (data not shown).

|  | Do you think that you understand the dangers of diabetes? | | P value |
| --- | --- | --- | --- |
|  | Yes | No |  |
| Total number | 41 | 9 |  |
| Mean scores | 6.78 | 4.11 | 0.028 |
| N (%) |  |  |  |
| 1 score | 0 (0.00) | 1 (11.11) |  |
| 2 scores | 0 (0.00) | 3 (33.33) |  |
| 3 scores | 1 (2.44) | 2 (22.22) |  |
| 4 scores | 4 (9.76) | 0 (0.00) |  |
| 5 scores | 5 (12.20) | 0 (0.00) |  |
| 6 scores | 4 (9.76) | 0 (0.00) |  |
| 7 scores | 6 (14.63) | 0 (0.00) |  |
| 8 scores | 21 (51.22) | 3 (33.33) |  |

Note: T-tests were performed for the pairwise comparison of independent samples.

| **Survey of patients’ perspectives for diabetes dangers** | | | | | |
| --- | --- | --- | --- | --- | --- |
| **Basic information** | | | | | |
| Name _________________ | | Gender □ Male □ Female | | Age ______ Years | |
| Educational level | □ Primary school and below | | □ Middle and high school | | □ College and above |
| Occupation | □ Responsible person at government agency, enterprise, or public sector institutions | | □ Clerk and associated personnel | | □ Professional and technical personnel |
|  | □ Production personnel in agriculture, forestry, animal husbandry, fisheries, or water conservancy industry | | □ Personnel in commercial and service industry | | □ Soldier |
|  | □ Operation and associated personnel of manufacturing or transport equipment | | □ Not otherwise specified | |  |
| Economic condition | □ Extreme poverty | | □ Poverty | | □ Subsistence level |
|  | □ Well-to-do | | □ Affluent | | □ Extremely affluent |
| Smoking (regular smoking, 1 or more cigarettes per day in the past three months) | | | | □ Yes □ No | |
| Drinking (beer [350 ml or more] or white liquor [50 g or more], three or more times per week in the past three months) | | | | □ Yes □ No | |
| Exercise (30 minutes or more, three or more times per week in the past three months) | | | | □ Yes □ No | |
| Diet (diabetes diet per physician directions or at least not over-eating) | | | | □ Yes □ No | |
| Diabetes history__________ Years | | | |  | |
|  |  |  |  |  |  |

| Status of diabetes control and related reasons | |
| --- | --- |
| Do you think that you understand the dangers of diabetes? □ yes □ no | |
| Please answer the following questions one by one: | |
| 1. Do you think that diabetes is terrible? | Yes [ ] No[ ] |
| 2. Do you know that patients with uncontrolled diabetes would suffer from life-threatening events? | Yes [ ] No[ ] |
| 3. Do you know that poorly controlled diabetes would suddenly lead to coma? | Yes [ ] No[ ] |
| 4. Do you know that poorly controlled diabetes would lead to blindness? | Yes [ ] No[ ] |
| 5. Do you know that poorly controlled diabetes would lead to limb dysmotility? | Yes [ ] No[ ] |
| 6. Do you know that poorly controlled diabetes would lead to kidney failure? | Yes [ ] No[ ] |
| 7. Do you know that poorly controlled diabetes increases the risk of heart attack? | Yes [ ] No[ ] |
| 8. Do you know that poorly controlled diabetes increases the risk of stroke? | Yes [ ] No[ ] |
| 9. Do you know that poorly controlled diabetes would lead to lower extremity ulcers or even amputations? | Yes [ ] No[ ] |
|  |  |

Number: ____
